# Salmon lice (*Lepeophtheirus salmonis*) showing varying emamectin benzoate susceptibilities differ in neuronal acetylcholine receptor and GABA-gated chloride channel mRNA expression

**DOI:** 10.1186/1471-2164-14-408

**Published:** 2013-06-18

**Authors:** Stephen N Carmichael, James E Bron, John B Taggart, Jacqueline H Ireland, Michaël Bekaert, Stewart TG Burgess, Philip J Skuce, Alasdair J Nisbet, Karim Gharbi, Armin Sturm

**Affiliations:** 1 Institute of Aquaculture, School of Natural Sciences, University of Stirling, Stirling, FK9 4LA, UK; 2 Moredun Research Institute, Pentlands Science Park, Bush Loan, Penicuik, Midlothian, EH26 0PZ, UK; 3 The GenePool, School of Biological Sciences, The University of Edinburgh, Edinburgh, EH9 3JT, UK

**Keywords:** Drug resistance, Sea lice, Avermectin, Ligand-gated chloride channel, Cys-loop receptor

## Abstract

**Background:**

Caligid copepods, also called sea lice, are fish ectoparasites, some species of which cause significant problems in the mariculture of salmon, where the annual cost of infection is in excess of €300 million globally. At present, caligid control on farms is mainly achieved using medicinal treatments. However, the continued use of a restricted number of medicine actives potentially favours the development of drug resistance. Here, we report transcriptional changes in a laboratory strain of the caligid *Lepeophtheirus salmonis* (Krøyer, 1837) that is moderately (~7-fold) resistant to the avermectin compound emamectin benzoate (EMB), a component of the anti-salmon louse agent SLICE® (Merck Animal Health).

**Results:**

Suppression subtractive hybridisation (SSH) was used to enrich transcripts differentially expressed between EMB-resistant (PT) and drug-susceptible (S) laboratory strains of *L. salmonis*. SSH libraries were subjected to 454 sequencing. Further *L. salmonis* transcript sequences were available as expressed sequence tags (EST) from GenBank. Contiguous sequences were generated from both SSH and EST sequences and annotated. Transcriptional responses in PT and S salmon lice were investigated using custom 15 K oligonucleotide microarrays designed using the above sequence resources. In the absence of EMB exposure, 359 targets differed in transcript abundance between the two strains, these genes being enriched for functions such as calcium ion binding, chitin metabolism and muscle structure. γ-aminobutyric acid (GABA)-gated chloride channel (GABA-Cl) and neuronal acetylcholine receptor (nAChR) subunits showed significantly lower transcript levels in PT lice compared to S lice. Using RT-qPCR, the decrease in mRNA levels was estimated at ~1.4-fold for GABA-Cl and ~2.8-fold for nAChR. Salmon lice from the PT strain showed few transcriptional responses following acute exposure (1 or 3 h) to 200 μg L^-1^ of EMB, a drug concentration tolerated by PT lice, but toxic for S lice.

**Conclusions:**

Avermectins are believed to exert their toxicity to invertebrates through interaction with glutamate-gated and GABA-gated chloride channels. Further potential drug targets include other Cys-loop ion channels such as nAChR. The present study demonstrates decreased transcript abundances of GABA-Cl and nAChR subunits in EMB-resistant salmon lice, suggesting their involvement in avermectin toxicity in caligids.

## Background

The development of synthetic insecticides and parasiticides has revolutionised the control of arthropod pests and parasites of agricultural, medical and veterinary importance. However, the overuse of chemical control agents tends to promote the development of heritable insecticide or drug resistance [1], with life-cycle traits of targeted organisms often favouring the ability to adapt genetically, such as high reproductive potential and short generation span. Drug resistance can follow from pre-existing mutations in resistance genes, termed resistance alleles, which under normal conditions are rare and have a negative or neutral effect on fitness but which, under conditions of exposure to control agents, afford fitness benefits and can become enriched in the target species’ gene pool given persisting selection pressure from the control agent [2]. By the time resistance becomes apparent as treatment failure, resistance alleles have usually already reached high frequencies in the gene pool [2].

Caligid copepods, also called sea lice, are common ectoparasites of marine fish [3]. One species, the salmon louse (*Lepeophtheirus salmonis* (Krøyer, 1837)), has emerged as a serious problem in mariculture of Atlantic salmon (*Salmo salar* Linnaeus, 1758) in the Northern hemisphere. The annual cost of sea louse infection to the global salmon farming industry has been estimated at €300 million, with the majority of this accounted for through expenses accrued from treatments with veterinary medicines [4]. Only a limited range of anti-sea louse drugs are available and licensed for the treatment of fish, and the continued use of a relatively small number of compounds creates a situation potentially favouring the development of drug resistance [5]. In the salmon louse, losses of efficacy have been reported for a number of control agents including organophosphates [6], pyrethroids [7], hydrogen peroxide [8] and avermectins (AVMs) [9,10].

The commonly used anti-sea louse treatment SLICE® (Merck Animal Health) contains the avermectin compound emamectin benzoate (EMB) (Stone et al., 1999). SLICE® is administered orally and a one-week treatment provides prolonged protection against all host-attached life stages of sea lice [11]. Avermectins are also used against external and internal parasites of humans and livestock, including parasitic nematodes causing the human diseases onchocerciasis (River blindness) and lymphatic filariasis, as well as gastrointestinal parasites of sheep, cows and horses [12]. The selective toxicity of avermectins against ecdysozoan invertebrates is believed to be based on the binding and blockage of glutamate-gated (GluCl) and γ-aminobutyric acid (GABA)-gated (GABA-Cl) chloride channels in the invertebrate nervous system [13]. Several molecular mechanisms have been suggested as contributing factors to the resistance of parasitic nematodes to the AVM compound ivermectin (IVM) [14]. Functional studies revealed that resistant nematodes can have single amino acid mutations in subunits of GluCl and GABA-Cl that decrease the channels’ sensitivities to the drug [15,16]. Furthermore, resistant nematodes may show increased expression of ABC (ATP-binding cassette) transporters, a group of membrane proteins with members capable of mediating the cellular efflux of drugs [17,18]. Finally, avermectin resistance in insects has been connected to alterations in drug metabolism [19].

Previous studies on potential molecular mechanisms of EMB resistance in salmon lice have used the candidate gene approach, *i.e.* the study of genes that have previously been linked to drug resistance in other organisms. In particular, such studies have investigated salmon louse ABC transporters [9,20] and GABA-Cl and GluCl subunits [21]. However, potential outcomes resulting from such candidate approaches are limited by the current knowledge of the biological process being studied. To avoid this limitation, a transcriptomic approach was followed in the present study, in which microarray analysis was used to compare mRNA responses between drug susceptible and moderately (~7-fold) EMB resistant laboratory strains of salmon lice.

## Results

### Experimental design

**Figure 1:**
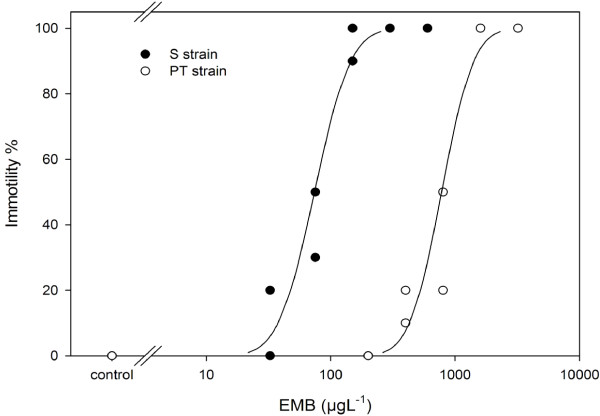
**Susceptibility of salmon louse laboratory strains to emamectin benzoate (EMB).** Toxicity responses of adult male salmon lice (*Lepeophtheirus salmonis*) laboratory strains S and PT in 24 hours immotility bioassays. Symbols represent the immotility response observed in one of duplicate beakers of ten individuals included for each combination of strain and treatment. Dose–response relationships (solid lines) were established using probit analysis, and yielded EC50 values of 73.9 μg L^-1^ (95% confidence intervals: 58.9 - 92.0 μg L^-1^) for the S strain and 642.3 μg L^-1^ (642.3 - 957.4 μg L^-1^) for the PT strain.

Two laboratory-maintained strains of salmon lice were used in the present study. Strain S is susceptible to all currently licensed anti-sea louse treatments including EMB, while strain PT is moderately resistant to EMB (Figure 1) [9]. Adult male salmon lice were used for the transcriptomic analyses as they are considered to provide a more steady physiological state than adult females, which undergo considerable morphological change following fertilisation and which are subject to repeated cycles of egg production. Salmon lice were collected from host fish anaesthetised in 100 mg L^-1^ 2-phenoxyethanol and were then allowed to recover in aerated seawater for 2 hours before use.

**Figure 2:**
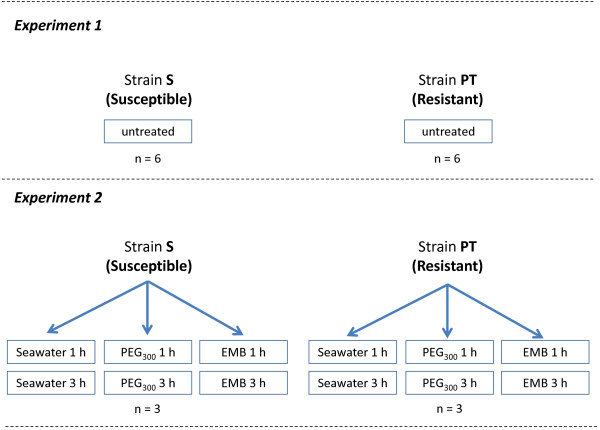
**Design of microarray experiments.** Microarray analysis was used to measure transcriptomic profiles in drug-susceptible (S) or EMB resistant (PT) salmon louse strains. In experiment 1, adult male salmon lice were collected for RNA extraction directly after removal from host fish. In experiment 2, salmon lice were removed from hosts and then subjected to exposure of seawater containing 200 μg L^-1^ of EMB for 1 or 3 hours before sampling. Control treatments included exposures to seawater, or seawater containing the solvent PEG_300_, which was used to solubilise EMB. The number of pools of four salmon lice sampled for each combination of treatment and strain is given as n.

In experiment 1, RNA expression profiles were analysed in lice sampled directly after recovery in order to reveal differences in constitutive gene expression between strains. Experiment 2 investigated the effects of short-term (1 to 3 hours) exposure to 200 μg L^-1^ of EMB, compared to seawater and carrier controls (Figure 2). This concentration of EMB resulted in >95% immotility of S lice after 24 hours, but had no observable effects on PT lice (Figure 1). We hypothesised that, should the tolerances of PT lice to EMB require transcriptional regulation, this should become apparent in transcriptomic profiles during the early stages (1 and 3 hours) of exposure. In addition to being investigated for effects of EMB, the data from experiment 2 were also analysed with respect to differences in constitutive transcript expression between strains. For this purpose, data for the one and three hour sea water controls from experiment 2 were pooled for each strain in order to obtain the same level of replication as used in experiment 1 (n = 6 biological replicates, *i.e.* pools of four *L. salmonis*). Control data from experiment 2 were pooled between 1 and 3 hour time points as we had previously observed that exposure of salmon lice to seawater for up to 12 hours after collection from the host has no effect on responses to EMB in water-borne bioassays (data not shown).

### Custom microarray design

For transcriptomic analyses, custom Agilent 15 K feature 60mer oligonucleotide microarrays were designed using sequences derived from salmon louse suppression subtractive hybridisation (SSH) libraries created in the present study together with expressed sequence tags (EST) publicly available in GenBank. Although the microarrays differed in a minor way between experiments 1 and 2 due to continuous elaboration/modification, the features included on the microarrays employed largely overlap (Table 1). To obtain a pool of salmon louse cDNA sequences enriched for transcripts differentially expressed between the EMB-resistant (PT) and drug-susceptible (S) salmon louse strains, two SSH libraries were constructed, corresponding to subtractions between strains in both directions. A pool of both libraries was subjected to Roche 454 sequencing, producing a total of 94,834 reads (N50 value of 289 nucleotides). The assembly of contiguous sequences (contigs) from sequence reads provided 1,916 annotated (BLASTx e-value <10^-4^) and 783 un-annotated target sequences. KEGG functional analysis of the annotated sequences revealed a large representation (53%) of genes involved in metabolism (data not shown). In addition, 129,225 *L. salmonis* ESTs (> 100 bp) were obtained from GenBank and assembled into contigs, providing a further 10,056 annotated (BLASTx e-value <10^-4^) and 2,526 un-annotated target sequences for the design of oligo probes to be used in the microarray designs (Table 1).

**Table 1:** **Composition of features on custom *L. salmonis *oligo microarrays**

**Probe type**	***Experiment 1 (AMADID # 039612)***	***Experiment 2 (AMADID # 033382)***
Annotated	13, 542	10,056
Unannotated	1,566*	5,052*
Control probes	100	100
Agilent controls	536	536
Total	15744	15744

* Oligo probes were designed to both the forward and reverse complement sequence for all targets that were not suitably annotated but included on the custom microarrays.

### Analysis of strain differences in constitutive gene expression

**Figure 3:**
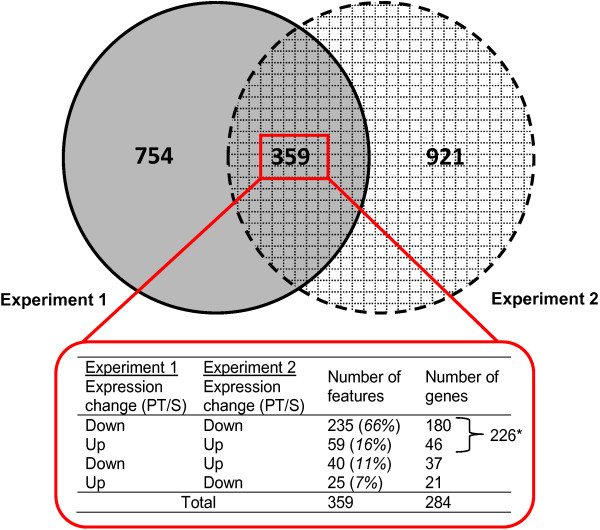
**Comparison of microarray features differentially expressed across salmon louse strains between experiments.** Genes differentially expressed between two salmon lice strains differing in EMB susceptibility (S, PT) were determined by microarray analysis in two independent experiments. Features that showed differential expression between strains (fold change ≥ 1.3, p < 0.05) were derived for both experiments. The Venn diagram only includes features studied in both experiments. *A total of 226 genes showed comparable differential expression between salmon louse strains in two independent microarray experiments and were therefore selected for further analysis of biological function.

To determine constitutive differences in gene expression between the PT and S strains, mRNA expression profiles were analysed in adult male salmon lice sampled in February 2012 (experiment 1) and May 2011 (seawater controls of experiment 2). When data from each experiment were analysed including only features present on both microarrays, similar numbers of features were found to be differentially expressed between strains in experiment 1 and experiment 2 (1,113 and 1,280 features respectively; Figure 3). Comparison of these two feature lists revealed that only 359 features were reported as being significantly differentially expressed between strains in both experiments. Of these, 294 (82%) showed the same direction of strain differences in the two experiments (Figure 3) and represented 226 genes of which 57% were annotated. These 226 genes were arranged by significance of the expression differences determined in experiment 1.

Genes that were represented in the top 100 most significantly differentially expressed transcripts are detailed in Table 2, which includes a substantial number of cytoskeleton proteins (26%) and proteases (12%). Enrichment analysis of the 294 features resulting from comparison of expression between strains was performed with respect to the gene ontology (GO) annotation representation on the microarray. Nine GO attributes were found to be significantly over-represented (Additional file 1), with calcium ion binding, structural constituent of muscle and actin binding being shown to be the most significantly over-represented GO terms.

**Table 2:** Genes showing differential expression between salmon louse strains differing in EMB susceptibility

**Accession no.**	**Annotation**	**Experiment 1**		**Experiment 2**	
		**Fold change (PT/S)**	**p-value**	**Fold change (PT/S)**	**p-value**
*Ligand gated ion channel (<1%)*					
ADD24187.1	Neuronal acetylcholine receptor subunit alpha-3	-3.21	7.59E-05	-2.52	4.29E-03
*Metabolism of xenobiotics (<1%)*					
ACO15001.1	Cytochrome P450 3A24	-1.48	1.95E-03	-1.31	4.13E-02
*Regulation of synapse development (<1%)*					
ADD24462.1	Cerebellin-3	-1.86	3.60E-04	-4.91	3.53E-06
*Eye lens proteins (<1%)*					
ADD38111.1	Beta-crystallin A1	2.33	6.79E-05	1.37	1.35E-02
*Transporters (2%)*					
NP_001116712.1	Solute carrier family 8 (sodium/calcium exchanger)	-2.10	3.63E-04	-4.38	1.74E-05
EFX88361.1	Alpha subunit of Na+/K+ ATPase	-1.67	2.21E-04	-2.08	5.34E-05
ACO12613.1	Excitatory amino acid transporter 3	-1.42	1.10E-04	-1.34	4.96E-02
*Cytoskeleton proteins (26%)*					
ADD38332.1	Troponin I	-1.77	4.85E-05	-2.61	1.17E-06
XP_001950563.1	Muscle LIM protein	-1.67	1.57E-04	-2.30	1.32E-06
AAA17371.1	Fast myosin heavy chain	-2.11	1.73E-03	-6.23	1.68E-06
ACO13186.1	Myosin light chain alkali	-2.65	1.69E-05	-3.38	8.46E-06
ACO12924.1	Myosin light chain alkali	-2.06	1.16E-05	-3.69	9.66E-06
ABU41018.1	Collagen alpha-1 chain	-1.38	1.51E-03	-6.85	2.61E-05
EFV61840.1	Smoothelin	-1.43	4.03E-04	-1.85	6.25E-05
ACO12887.1	Troponin T	-1.73	1.04E-03	-2.93	8.12E-05
ADV40202.1	Troponin 1	-1.94	1.87E-03	-2.93	3.23E-04
AAW22542.1	Myosin light chain	-1.76	1.00E-03	-2.23	3.73E-04
ACO10528.1	Troponin C, isoform 1	-1.91	1.69E-03	-2.22	5.12E-04
ACO12630.1	Troponin C, isoform 1	-1.61	3.01E-05	-1.51	5.92E-04
ACO12421.1	Tropomodulin	-1.51	1.17E-04	-2.37	1.01E-03
ACO12794.1	Troponin C, isoform 1	-2.07	6.14E-05	-1.60	2.85E-03
ACO14751.1	Troponin C, isoform 1	-2.12	4.94E-05	-1.56	4.18E-03
ACO11818.1	Torso-like protein precursor	-1.83	5.11E-05	-1.82	9.67E-03
ACO11077.1	Troponin C, isoform 1	-1.92	6.27E-05	-1.48	1.56E-02
*Regulation of actin cytoskeleton (<1%)*					
XP_002407362.1	Paxillin	-2.79	8.32E-07	-1.48	2.28E-03
*Calcium transport (<1%)*					
NP_001032719.1	Sarco/endoplasmic reticulum calcium transporting ATPase	-2.15	8.58E-07	-2.46	2.15E-04
*Calcium binding (1%)*					
XP_002734090.1	Calmodulin-like	-1.60	6.41E-05	-2.46	3.25E-04
ACO11757.1	Sarcoplasmic calcium-binding protein, beta chain	-3.18	2.05E-06	-3.58	1.28E-03
*Cuticle proteins (4%)*					
ADD24515.1	Cuticle protein 6	-2.29	1.79E-03	-12.28	4.75E-04
ABU41025.1	Cuticle protein	-2.57	2.78E-04	-12.91	1.88E-03
ACO14885.1	Cuticle protein CP14.6 precursor	-1.77	7.50E-04	-3.40	4.67E-03
*Proteolysis (12%)*					
ADD38666.1	Matrix metalloproteinase-9	-2.29	1.07E-05	-7.35	1.84E-06
ADD38283.1	Kunitz/BPTI-like toxin	-2.45	5.43E-06	-1.79	2.50E-04
ABU41053.1	Metalloproteinase	-5.61	7.00E-06	-4.38	3.99E-04
BAG74353.1	Metalloproteinase	14.83	8.20E-05	18.61	4.64E-04
ACO11096.1	Serine carboxypeptidase CPVL precursor	1.58	2.44E-04	1.36	1.21E-02
ABU41117.1	Metalloproteinase	-2.72	6.62E-05	-1.41	1.82E-02
AAS91793.1	Intestinal trypsin 2 precursor	7.73	1.90E-06	1.82	2.52E-02
AAS91795.1	Intestinal trypsin 4 precursor	1.48	6.69E-04	1.42	2.79E-02

Annotated genes (57%) were sorted by the significance of differential expression between strains in experiment 1 and arranged by biological function. Features with identical annotation were removed prior to categorising biological function. Also indicated is the percentage representation for each functional category in the total number of annotated genes (129 genes).

To confirm findings from microarray analyses, transcript abundance was analysed for a sub-set of significantly differentially expressed genes using RT-qPCR. Genes were selected on the basis of potential significance as pharmacological targets of EMB (GABA-Cl subunit alpha and neuronal acetylcholine receptor subunit α3), or detoxification mechanisms (cytochrome P450 isoforms, carboxylesterase). Maltase-glucoamylase was further included because of its high level of differential expression (105-fold) between salmon louse strains. RT-qPCR analysis found that transcripts of nAChR α-3 were ~3.1-fold and ~2.6-fold less abundant in the PT than the S strain in experiments 1 and 2 respectively, which confirmed trends observed in the microarray analyses (Table 3). Similarly, RT-qPCR demonstrated that PT lice showed significantly lower levels of GABA-Cl α-subunit mRNA expression compared to the S strain (1.4-fold and 1.6-fold in experiments 1 and 2, respectively; Table 3), although differences were marginal. Isoforms of cytochrome P450 and carboxylesterase, *i.e.* enzymes potentially involved in detoxification, were found to show higher mRNA expression levels in the PT compared to the S strain in experiment 1, but not experiment 2. Transcript levels of maltase-glucoamylase were much lower in PT than S lice in experiment 1, whereas in experiment 2 the mRNA expression was moderately increased in PT compared to S lice (Table 3).

**Table 3:** Gene expression measured by RT-qPCR in salmon lice from two strains differing in EMB susceptibility

**Accession no.**	**Annotation**	**Experiment 1**						**Experiment 2**					
		**Microarray**		**RT-qPCR**		**Pearson correlation**		**Microarray**		**RT-qPCR**		**Pearson correlation**	
		**p-value**	**Fold change (PT/S)**	**p-value**	**Fold change (PT/S)**	**r**	**p-value**	**p-value**	**Fold change (PT/S)**	**p-value**	**Fold change (PT/S)**	**r**	**p-value**
ADD24187.1	Neuronal acetylcholine receptor subunit α3	7.59E-05	-3.21	0.000	-3.08	0.93	<0.0001	4.29E-03	-2.52	0.009	-2.64	0.89	0.0001
EFN73916.1	GABA receptor subunit alpha	NS	-1.19	0.015	-1.35	N/A	N/A	2.05E-04	-1.75	0.005	-1.56	0.89	0.0001
XP_003494528.1	Cytochrome p450 18a1	3.42E-08	3.34	0.000	3.14	0.95	<0.0001	N/A		NS	1.00	N/A	N/A
AAS13464.1	Cytochrome p450 15a1	8.93E-04	2.14	0.000	1.82	0.95	<0.0001	N/A		NS	1.09	N/A	N/A
NP_001136104.1	Carboxylesterase	1.13E-04	1.38	0.041	1.21	0.65	0.02	NS	1.12	NS	1.12	N/A	N/A
XP_797271.2	Maltase-glucoamylase	9.12E-05	-104.50	0.000	-194.85	0.99	<0.0001	7.10E-03	1.72	0.017	2.00		

Abbreviations: *NS* Not significantly different (p<0.05), *N/A* Not applicable.

For selected genes, mRNA expression was measured in salmon lice from two strains differing in EMB susceptibility (see Figure 1 for information on strains), results compared to findings obtained in microarray analyses. Significance (p < 0.05) assessed by *t*-test (Welch) for microarray analysis and one-way ANOVA for RT-qPCR analysis. Fold changes ≥ 1.3 are underlined.

### Effects of short-term exposure to EMB on transcript profiles in salmon lice

**Figure 4:**
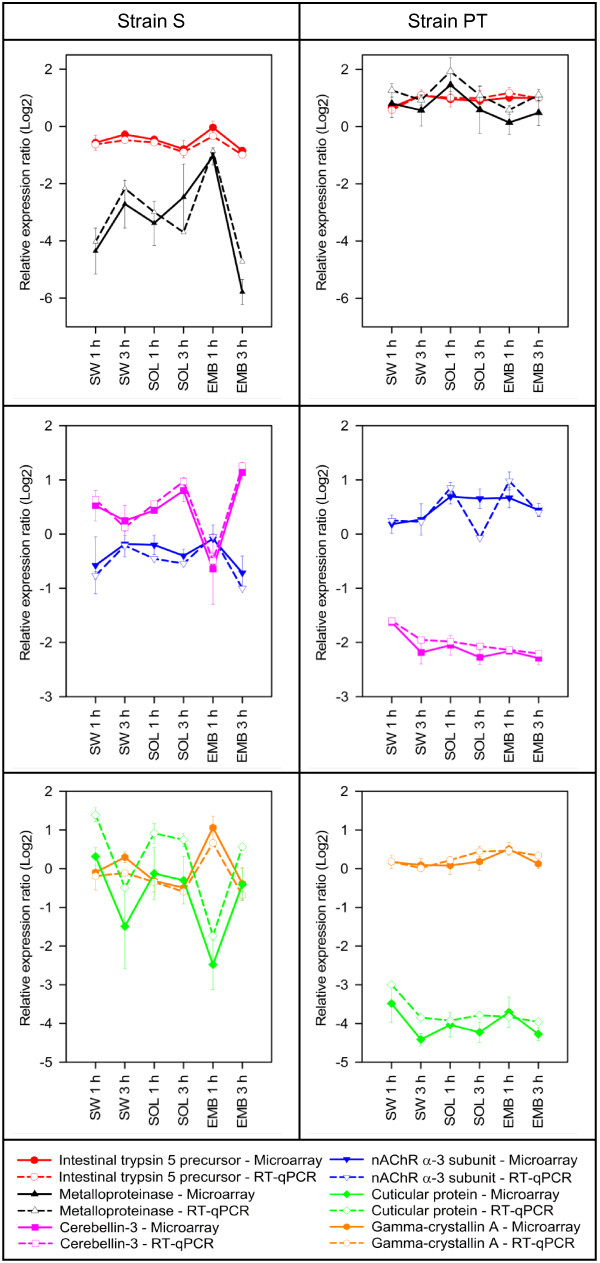
**Gene expression responses at early time points of exposure to EMB.** Shown are the relative expression ratios (RER) measured by microarray and RT-qPCR analysis of S and PT salmon louse strains after 1 and 3 hours exposure to 200 μg L^-1^ EMB, seawater (SW), or the solvent PEG_300_ (SOL) . Data are Log_2_ RER ± SE (n = 3).

Three-way ANOVA of the microarray expression data from experiment 2 showed that a large proportion (55%) of the total number of features that passed quality filtering (n = 10,804) was affected by the factor strain. In contrast, the factor treatment had a comparatively small influence on gene expression (Table 4). To confirm these microarray results, transcript abundances were determined by RT-qPCR for six selected genes. Genes were selected so as to include a number of qualitatively different expression profiles, detectable fold-changes and selected candidate genes (GABA-α and AChR-α3 subunits) (Figure 4). A high degree of correlation was observed between expression values measured by both methods (Pearson correlation coefficients (r) of 0.71 to 0.99; p <0.0001).

**Table 4:** Number of differentially expressed features identified in microarray analysis of samples from experiment 2

**Experimental factor**	**Number of features**
Strain	5940 (55%)
Treatment	369 (3%)
Time	968 (9%)
Strain x Treatment	406 (4%)
Strain x Time	950 (9%)
Time x Treatment	1309 (12%)
Strain x Treatment x Time	1701 (16%)

Significance (p-value < 0.05) assessed by three-way ANOVA. Also indicated is the percentage of the total number of features (10, 804) used in the analysis.

**Figure 5:**
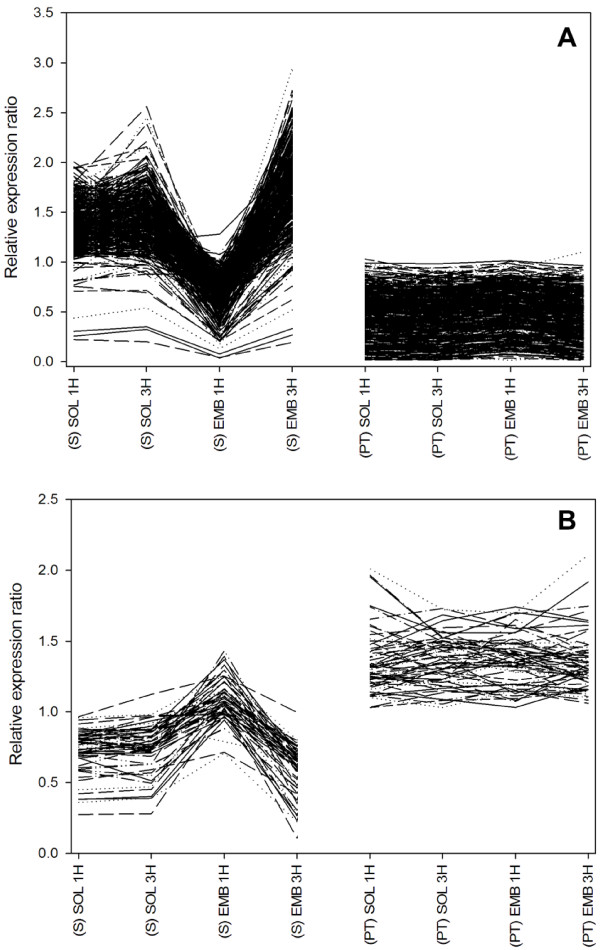
**Relative expression profiles for the features in network clusters one (A) and two (B).** A total of 418 (cluster 1) and 62 (Cluster 2) features (fold change ≥ 1.3 in S strain) were clustered using network analysis. The similarity of expression profiles were measured using the Pearson correlation coefficient and clustered using the Markov clustering algorithm (MCL).

To further investigate the effects of EMB exposure, a list of those features that were significantly affected by EMB treatment or for which significant interactions between treatment and other factors were observed (treatment × strain; treatment × time; treatment × strain × time) was compiled. This list comprised a total of 2,020 features, of which at least 35% were involved in metabolism (KEGG functional classification, data not shown). Transcript abundance profiles for the 2,020 features were further subjected to network clustering using the BioLayout Express^3D^ application [22]. This resulted in the resolution of 59 clusters with a minimum cluster size of four features. The two main clusters 1 and 2 contained 418 and 62 features, respectively, that showed fold changes > 1.3 across all conditions (Figure 5A & B). Within the two clusters, expression profiles were characterised by pronounced responses in S lice following 1 h of EMB exposure (down-regulation in cluster 1, up-regulation in cluster 2), and a full or partial return to basal expression levels after 3 h of EMB exposure. Moreover, for genes in both clusters, few if any responses to EMB exposure were observed in the PT strain.

The most significantly differentially regulated genes from cluster 1, and the responses to EMB exposure in both salmon louse strains are summarised in Additional file 2. An analogous selection of genes from cluster 2 is provided in Additional file 3. Genes in cluster one include the GABA-Cl and nAChR α-3 subunits which are potential targets for EMB, with nAChR α-3 also having been identified as constitutively differentially expressed between salmon louse strains. Genes in cluster two included a glutathione-S-transferase isoform and a nAChR α-3 precursor. Enrichment analysis of the features in cluster one is detailed in Additional file 4, showing significant over representation of twelve and under representation of four GO attributes. Chitin binding, calcium ion binding and hydrolase activity were the most significantly over-represented attributes and nucleic acid binding was identified as the most significant under represented attribute.

## Discussion

Using transcriptional profiling in comparative studies of a drug-susceptible and an EMB-resistant salmon louse strain, this study demonstrated the reduced constitutive mRNA expression of subunits of certain ligand-gated ion channels (LGIC) in the EMB resistant strain, namely a GABA-gated chloride channel subunit (GABA-Cl, ~1.4-fold decreased) and a neuronal acetylcholine receptor subunit (nAChR α-3; ~2.8-fold decreased). The toxicity of EMB and other AVMs against ecdysozoan invertebrates is reported to be based mainly on their interaction with another class of LGICs, the glutamate-gated chloride channels (GluCl) [23-25] although GABA-Cls are also believed to be pharmacological targets of AVMs [26]. While nAChRs are traditionally not considered to be implicated in the toxic action of AVMs in ecdysozoans, they can be allosterically modulated by IVM [27]. This study’s finding that mRNA levels of GABA-Cl and nAChR subunits are decreased in EMB-resistant salmon lice suggests that these LGICs may represent potentially additional target sites for AVMs in sea lice. When transcriptomic profiles of EMB-resistant and -susceptible laboratory salmon louse strains were investigated in response to short term (1–3 h) aqueous EMB exposure, a number of transcriptional responses to the treatment were observed in the drug-susceptible S lice, but few responses were found in the EMB-resistant PT strain. While we cannot exclude the possibility that EMB exposure might provoke more pronounced transcriptomic responses in PT lice at later time points, differential toxicity of EMB between the louse strains is apparent as early as 5 hours post-exposure (data not shown). This suggests that the mechanism of resistance, whether constitutive, responsive or a combination of both, must have occurred before this time point. The present study only considered levels of mRNA. Molecular mechanisms underlying differential susceptibilities between the studied strains could also include post-transcriptional regulatory mechanisms, such as mRNA processing and degradation, translation and protein degradation [28], however, these lay outside the scope of the present study.

AVMs are used against parasitic and pest species of ecdysozoan invertebrates including nematodes, insects and mites, and more recently against crustaceans. While the molecular target sites of AVMs in crustaceans are unknown, GluCls are generally considered to be the main pharmacological targets of IVMs in nematodes and insects [29,30]. The GluCls form an invertebrate-specific subgroup of the large ‘Cys-loop’ subfamily of LGIC. ‘Cys-loop’ LGICs have a pentameric structure, and are composed of either the same type of subunits or two to three different subunit isoforms. Consistent with the role of GluCl as the main target of AVMs, IVM-resistant strains of invertebrates can show mutations changing the expression levels or the peptide sequence of channel subunits [23,24]. A GluClα subunit has been cloned in *L. salmonis*[21] and while GluClα was represented amongst the microarray targets used for this study, no difference in mRNA expression was observed between salmon lice of the two studied strains, or amongst those subjected to control and sublethal EMB treatments (data not shown).

Apart from GluCl, further LGICs are known to interact with AVMs. For instance, IVM modulates the activity of nematode GABA-Cl [26], and can exert directly activating or potentiating effects on vertebrate glycine-gated chloride channels [31]. Moreover, AVMs can modulate the activity of cation-LGICs such as the α-7 nAChR [27] and the ATP-gated P2X_4_ receptors [32]. A number of observations involving drug-resistant insects and nematodes support the hypothesis that LGICs other than GluCl constitute further toxicologically relevant targets of AVMs in invertebrates. Cyclodiene-resistant fruit flies having a single amino acid mutation in a GABA-Cl showed a moderate degree of cross-resistance to IVM [24]. A null mutation in a histamine-gated chloride channel also conferred moderate IVM resistance in *Drosophila melanogaster* Meigen, 1830 [33], and a novel dopamine-gated ion channel (HcGGR3) was significantly down-regulated in an AVM-selected strain of the nematode *Haemonchus contortus* (Rudolphi, 1803) [34]. The observation in this study that EMB-resistant salmon lice show decreased mRNA levels of nAChR and GABA-Cl is consistent with findings in the literature cited above, and suggests a role for nAChR and GABA-Cl as additional pharmacological targets of EMB in salmon lice. It is worth noting in this context that observed changes in nAChR expression could also relate to previous exposure of PT lice to compounds interfering with cholinergic neurotransmission such as the organophosphate (OP) anti-sea louse drug azamethiphos [35]. However, decreases in nAChR expression are not among typical molecular responses associated with OP resistance in insects [1,36]. While decreased expression of nAChR has been observed in nematodes resistant to imidazothiazoles and other drugs that directly target nAChR channels [14], these are classes of compound that have not been used against sea lice.

Apart from modifications of the molecular targets, biocide resistance in pests and parasites can result from increased elimination of the chemical as a result of up-regulation of biotransformation enzymes and/or drug transporters. The superfamily of cytochrome P450s (CYPs) contains heme-thiolate proteins that function as monooxygenases, many of which are involved in drug metabolism [37,38]. CYPs play important roles as chemical resistance genes in insects [1,39], but their roles in the biochemical defence against toxicants in crustaceans are less well understood. The microarrays used in the present study included probes representing a number of target sequences that are annotated as CYPs (experiment 1: 18 probes; experiment 2: 14 probes). These partial *L. salmonis* CYP sequences could not be unequivocally attributed to specific CYP families, as this would require establishing the overall degree of amino acid similarity to CYP family members [40]. However, based on BLASTx annotation (e-values of ≤ 10^-7^), most of these sequences could be provisionally allocated to CYP clans, which are higher-order groupings that combine phylogenetically related CYP families [40] (Clan 2: 6 sequences; clan 3: 5 sequences; mitochondrial clan: 3 sequences). Two targets showing similarity to CYP15A1 and CYP18A1 (both clan 2) differed in mRNA expression in *L. salmonis* from experiment 1, but not experiment 2. Moreover, compared to the EMB susceptible strain a target showing similarity to CYP3A24 (clan 3) had lower expression levels in the EMB resistant strain, with a moderate (1.48-fold) difference in transcript abundance found between the strains. In insects, CYP15A1 is involved in juvenile hormone synthesis [41], whereas CYP18A1 functions to inactivate ecdysteroids [42]. Crustaceans possess homologues to both these clan 2 CYPs [43], but little is known of their function. In the green shore crab (*Carcinus maenas*) expression levels of two CYPs from clan 2 were affected by both the moulting cycle and previous exposure to xenobiotics [44]. The differences in CYP mRNA levels found in this study were relatively small and/or variable between experiments and therefore do not provide clear evidence for an involvement of CYPs in the differential EMB susceptibility found in the salmon louse strains that were studied.

Carboxylesterases are another class of enzymes that can confer insecticide resistance [1]. In this study, the expression of one carboxylesterase was moderately enhanced in EMB-resistant salmon lice in experiment 1, but no significant differences in expression were observed between strains in experiment 2. Accordingly, the data provides no evidence for a role of carboxylesterase in EMB resistance of salmon lice.

ABC (ATP-binding cassette) proteins are a family of membrane-bound transporters mediating the transport of a diverse array of substrates across biological membranes [45]. Certain ABC proteins are drug efflux transporters located in the cell membrane, and have roles in the biochemical defence against toxicants [46]. The ABC transporter P-glycoprotein transports IVM [47] and has relevance as a biochemical factor limiting the drug’s toxicity in mice and nematodes [18,48]. It has been suggested that P-glycoprotein could be implicated in the resistance of pests and parasites to AVMs [49-51], and a salmon louse homologue of P-glycoprotein called SL-PGY1 has been cloned [9]. In the present study, mRNA expression of SL-PGY1 was unaffected by EMB treatment and did not differ between S and PT strains (data not shown), confirming similar data previously reported for these strains [9].

In addition to comparing transcriptional profiles between louse strains in the absence of drug exposure, the present study also addresses transcriptomic responses of both louse strains to short-term exposures (1 to 3 hours) to 200 μg L^-1^ of EMB. The selected EMB concentration is not toxic to the resistant salmon louse strain, but in the drug-susceptible strain it results in close to 100% immotility after 24 hours exposure. After 1 to 3 hours of exposure, few transcriptomic responses were observed in the resistant strain, and therefore these data do not provide evidence for the presence of specific resistance mechanisms requiring transcriptional regulation. In the drug-susceptible strain, a complex array of transcriptional responses to EMB was observed. This finding was expected, as this level of EMB exposure was known to result in significant toxic responses after 24 hours. Early transcriptional responses are therefore likely to comprise both adaptive and general stress responses, as well as transcriptional changes reflecting the toxic action of the drug.

## Conclusions

The transcriptional profiling of a drug susceptible and an EMB resistant laboratory strain of salmon lice revealed reduced mRNA expression of a number of LGIC subunits in the EMB resistant lice. GluCl, considered to be the major target site of AVM drugs in invertebrates, displayed similar mRNA expression levels in EMB-resistant and reference strains. In contrast, subunits of GABA-Cl and nAChR showed decreased mRNA abundances in the EMB resistant compared to the reference strain. While GABA-Cl is considered a secondary target of AVMs in invertebrates, nAChR is not traditionally considered a target site for AVMs, even though it has been shown to interact with AVMs in vertebrates. It is possible that nAChR and GABA-Cl represent additional EMB target sites in salmon lice, and that the down-regulation of these channel subunits in this EMB-resistant strain could be related to the resistance phenotype. In the present study no changes were seen in the expression levels of biotransformation enzymes and drug transporters, both of which classes have been suggested to contribute to AVM resistance in other species. Further studies are needed to investigate potential relationships between the transcriptional changes observed and the susceptibility phenotype.

## Methods

### Salmon louse strains

Two laboratory-maintained salmon louse (*L. salmonis*) strains differing in susceptibility to EMB [9], were used in this study. The susceptible strain S was established in 2003 using salmon lice from a Scottish farm site where no chemical control agents other than hydrogen peroxide had been used. The moderately EMB-resistant salmon louse strain PT was established in December 2008 using salmon lice from another Scottish production site with reports of variable EMB treatment efficacies. The strains have since been cultured under identical laboratory conditions, as described in detail elsewhere [9]. In brief, salmon lice were maintained on Atlantic salmon (*S. salar*) with an initial weight of 500–1000 g in circular tanks supplied with fresh seawater at ambient temperature, using a photoperiod corresponding to natural day length. To propagate salmon louse cultures, egg strings were allowed to hatch and develop to copepodids, which were used to inoculate a tank containing fresh host fish. Prior to the collection of salmon lice from hosts, fish were anaesthetised with 100 mg L^-1^ 2-phenoxyethanol. Infection rates were maintained at levels that were unlikely to compromise fish welfare. All laboratory infections were carried out under UK Home Office licence and appropriate veterinary supervision.

### Salmon louse exposure experiments

Adult male salmon lice were collected from anaesthetised host fish as described above and allowed to recover for 2 hours in aerated filtered seawater at ambient sea temperature. To analyse transcript expression in salmon louse strains S and PT in the absence of drug exposure (microarray experiment 1), adult males were collected and preserved in an RNA stabilisation solution (4.54 M ammonium sulphate, 25 mM trisodium citrate, 20 mM EDTA, pH 5.4) prior to storage at -70°C. For both strains six pooled samples comprising four individuals each were taken. The effects of acute seawater-borne exposure to EMB on transcript expression were investigated in microarray experiment 2. In order to establish the EMB concentration for this experiment, 24 hour bioassays [52] were carried out on salmon louse strains S and PT. EMB (technical grade, a gift from Merck Animal Health) was solubilised in seawater with PEG_300_ (final concentration 0.01% (v/v)). EMB concentrations used in bioassays were 32.5, 75, 150, 300 and 600 μg L^-1^ with S lice, and 200, 400, 800, 1,600 and 3,200 μg L^-1^ with PT lice. Duplicate glass dishes containing 10 salmon lice and 200 mL of exposure solution were used per EMB concentration, control (seawater) or solvent control (seawater with 0.01% (v/v) PEG_300_). At the end of 24 hours of exposure, salmon lice were recorded as normally motile or immotile upon visual examination and stimulation with a fine brush. To generate samples for microarray experiment 2, salmon lice of either strain were subjected to short term (1 and 3 hour) exposures to 200 μg L^-1^ EMB, a concentration that would result in >95% immotility in S lice after 24 hours but have no effects in PT lice. In addition, the experiment comprised seawater and solvent (0.01% (v/v) PEG_300_) controls. For each combination of strain, exposure period and treatment, three pooled samples consisting of four salmon lice each were collected for later RNA extraction as above. None of the treatments had effects on louse motility. At the end of the experiment (3 hours), water samples were taken and sent to a commercial laboratory (Eclipse Scientific, Chatteris, UK) for EMB residue analysis (liquid chromatography with detection by MSMS). The measured EMB concentration in the nominal 200 μg L^-1^ EMB treatment was 99.5 ± 5.2 μg L^-1^ EMB. This depletion of solubilised active ingredient may be attributed to EMB adsorption to the glass containers used for exposure assays [53].

### RNA extraction and purification

In microarray and RT-qPCR experiments, samples were pools of four adult male salmon lice. Frozen samples were ground in liquid nitrogen using a pestle and mortar, and total RNA was immediately extracted from the homogenised sample using TRI Reagent® (Sigma-Aldrich, UK), following the manufacturer’s protocol. After phase separation, RNA was precipitated from the aqueous phase by addition of 0.25 volumes isopropanol and 0.25 volumes of a high salt buffer (0.8 M trisodium citrate; 1.2 M sodium chloride), as recommended for samples with high polysaccharide content [54]. The total RNA was resuspended in nuclease-free water and further purified using RNeasy columns (Qiagen, UK). For the construction of subtracted cDNA libraries, total RNA from 60 untreated adult males from either strain (S or PT) were pooled and subjected to poly (A)+ RNA isolation using the Poly (A) Purist™ kit (Ambion®, UK). UV spectroscopy (NanoDrop ND-1000, Thermo Scientific, USA) was used to confirm purity of the RNA samples and establish concentrations, whereas RNA integrity was assessed by agarose gel electrophoresis and ethidium bromide staining.

### Subtracted cDNA library construction and sequencing

Suppression subtractive hybridisation (SSH) was used to prepare cDNA libraries enriched in transcripts differentially expressed between strains S and PT using commercial methods (PCR-Select™, Clontech, Takara Bio Inc., USA) . Subtractions were performed in both directions, *i.e.* using cDNA derived from each strain (S or PT) either as the tester or the driver. A pool of cDNA from each subtraction, containing an equal amount of both subtracted cDNA libraries, was used for generating a 454 sequencing library using the GS FLX Titanium Rapid Library Preparation kit (Roche Applied Science, UK), following manufacturer’s instructions. Adaptive Focus Acoustics™ (AFA™) using the S220 High Performance Ultrasonicator (Covaris® Inc., KBiosciences, UK) was employed to randomly shear the cDNA, blunt ends were repaired and MID adapters ligated to the DNA fragments prior to sequencing using the Genome Sequencer™ (GS) Titanium FLX instrument (Roche Applied Science, UK) (EBI Sequence Read Archive (SRA) study ERP002190). GS FLX Titanium library preparation and sequencing was performed by The GenePool Genomics Facility (University of Edinburgh, UK). Sequence reads were assembled using the GS De Novo Assembler (Newbler) v2.5.3 software (Roche Applied Science, UK) using default parameters after trimming of MID adapter and primer sequences.

### Sequence assembly from existing *L. salmonis* EST resource

A total of 129,225 sequences (> 100 bp) were downloaded during December 2010 for *L. salmonis* from the GenBank EST database (as described by Yasuike et al.) [55], and assembled into contigs using default assembly settings of The Gene Indices Clustering Tools (TGICL), obtained from the Computational Biology and Functional Genomics Laboratory (The Gene Index Project, USA). Prior to sequence assembly, vector sequences were removed using SeqMan II 6.1 (DNAStar Inc., USA).

### Salmon louse microarray design

The assembled contig sequences were annotated using BLASTx (Basic Local Alignment Search Tool) searches against the non-redundant proteins (nr), UniprotKB/Swiss-Prot (Swissprot) and Reference Proteins (Refseq_Proteins) GenBank databases at the National Centre for Biotechnology Information (NCBI), with an annotation hit having an expectation value (e-value) of <1 × 10^-4^ being considered significant. All sequences were further annotated with GO identifiers using Blast2Go software for Windows® using Java Webstart (Centro de Investigación Príncipe Felipe, Spain). Oligonucleotide probes (60mers) were designed to target contig sequences using the eArray Gene Expression (GE) probe design tool (Agilent Technologies, UK), employing the base composition and best probe methodologies, and designed in sense orientation with 3’ bias. For each sequence without a significant BLASTx based annotation two probes were selected; designed to both forward and reverse complement sequences. Standard expression microarrays were designed using the eArray custom microarray design wizard (Agilent Technologies, UK) for an 8 × 15 K design format. Each microarray comprised 15,744 features including 536 obligatory controls (Table 1). An initial design was used for experiment 1 interrogations (Agilent AMADID No 039612; EBI ArrayExpress design A-MEXP-2285). This design incorporated probes designed to target 2,699 sequences that were identified when sequencing the subtracted cDNA libraries enriched for transcripts differentially expressed between the EMB-resistant (PT) and drug susceptible (S) salmon louse strains. Two probes were designed for each of the SSH targets. Experiment 2 employed a modified design (Agilent AMADID No 033382; EBI ArrayExpress design AMEXP-2284). The array designs shared 10,251 identical features.

### Microarray analyses

Labelling protocols are described in detail elsewhere [56]. Briefly, for each test sample 250 ng total RNA was used as template for the amplification of antisense RNA with the incorporation of the modified nucleotide 5-(3-aminoallyl)-UTP (aaUTP) into the amplified RNA (aRNA) during the *in vitro* transcription step (TargetAmp™ Aminoallyl-aRNA Amplification Kit 101; Epicentre®, Cambio Ltd. UK). A common reference pool was created through pooling equal amounts of all aRNA test samples to be used in the experiment. The individual test samples were labelled with cyanine 3 (Cy3) and the common reference pool labelled with Cy5 mono-reactive dye (GE Healthcare, UK) in dye coupling reactions. Unincorporated dye was removed by column purification (Illustra Autoseq™ G-50 spin columns; GE healthcare, UK), and then dye incorporation was assessed by spectrophotometry (NanoDrop ND-1000, Thermo Scientific, USA) and fluorescent gel electrophoresis. Three hundred nanograms of each Cy3-labelled test sample was competitively hybridised with 300 ng Cy5-labelled common reference pool on a 15 K feature custom microarray, following the manufacturer’s instructions (Agilent Technologies, UK). The hybridisation reactions were incubated at 65°C with 10 rpm rotation for 17 hours in an Agilent rotary hybridisation oven and then washed with Gene Expression Wash Buffers 1 and 2, with a final wash using Stabilisation and Drying solution, again following the manufacturer’s instructions (Agilent Technologies, UK). The hybridised microarrays were scanned using an Axon Genepix 4200A scanner with Genepix Pro 6.1 image acquisition software (Molecular Devices, UK) using 40% laser power, 5 μm pixel size resolution and auto photo-multiplier tube (auto-PMT) function with 0.05 saturation tolerances. The raw microarray images were processed using Agilent Feature Extraction (FE) software version 9.5.3.1 that performed feature grid alignment, extraction and quantification. The fluorescence intensity results files from the FE software were imported into the GeneSpring GX version 12 software (Agilent Technologies, UK) for differential gene expression analysis. Data were normalised using Lowess normalisation of log_2_-expression ratios without baseline transformation. Features showing low quality according to Agilent quality control metrics were discarded to provide a final feature set for analysis. Details of microarray experiments 1 and 2 have been submitted to ArrayExpress and assigned accession numbers E-MTAB-1484 (Experiment 1) and E-MTAB-1478 (Experiment 2). The recording of the microarray experimental metadata complies with Minimum Information About a Microarray Experiment (MIAME) guidelines.

### RT-qPCR

To validate gene expression results from microarray experiments, the abundance of selected differentially expressed transcripts was determined by reverse transcription quantitative PCR (RT-qPCR). Per experiment, three targets that showed stable expression levels in microarray hybridisations were selected as reference genes. (Experiment 1: 60S ribosomal protein S20, 40S ribosomal protein L44 and RMD-5 homolog; experiment 2: Hypoxanthine-guanine phosphoribosyltransferase (HGPRT), RMD-5 homolog and Elongation factor 1α). For each target sequence, primers were designed with a melting temperature (Tm) of ~60°C using Primer 3 software (Additional file 5). Aliquots (1 μg) of total RNA samples previously used in microarray analyses were reverse transcribed (Superscript III, Invitrogen, UK) using random hexamers and anchored oligo-dT in a 3:1 molar ratio. No-template controls and controls omitting RT enzyme were included on each assay plate to detect potential DNA contamination. A cDNA pool containing equal amounts of all samples was made and included on each assay plate, serving as a calibration sample (20-fold dilution) and for derivation of a standard curve from serial dilutions. RT-qPCR reactions were performed in duplicate in a total volume of 20 μL containing 5 μL sample cDNA (20-fold dilution), 0.3 μM of each primer and 10 μL Absolute SYBR Green I mix (ThermoFisher Scientific, UK), using the Mastercycler ep realplex^2^ (Eppendorf, UK) with the following amplification conditions: 95°C for 15 minutes, followed by 40 cycles of 94°C for 30 seconds, 15 seconds at the specific primer pair annealing temperature (T_a_; Additional file 5) and 72°C for 30 seconds. After amplification a melt curve from 55°C to 95°C at 0.5°C increments for 15 seconds each was performed to ensure that a single product was amplified in each reaction. Threshold cycles were analysed using the PCR cycler software. Standard curves were derived from plots of the threshold cycle against the logarithm of the relative concentration of cDNA pool. Primer efficiency (E) was derived from linear fits to the standard curve according to the equation E = 10^(-1/slope)^. The BestKeeper tool [57] was employed to analyse expression stability of three reference genes and determine a robust BestKeeper expression index as a geometric mean for the three reference genes, which was in turn used to establish relative gene expression ratios using the ΔΔCt method using the Relative Expression Software Tool (REST) Multiple Condition Solver (MCS) [58].

### Statistical analysis

Microarray gene expression data were analysed using GeneSpring GX version 12 (Agilent Technologies). The analysis of constitutive differential gene expression in experiments 1 and 2 used Student’s *t*-test adapted for samples with unequal variance (Welch) using a fold change threshold of 1.3. The analysis of differential gene expression induced in microarray experiment 2 employed two-way ANOVA to compare exposure of both salmon louse strains at two time points against control conditions. Multiple testing corrections were not applied to any statistical analysis of this gene expression study as this can often be over-conservative when studying potentially subtle gene expression responses to stimuli [56,59]. This decision is supported by confirmation of differential expression by RT-qPCR in the current study. Network analysis of microarray experiment 2 expression data was performed using the BioLayout Express^3D^ application [22]. A network graph was constructed using the Pearson correlation coefficient (threshold of 0.94) to determine similarities between expression profiles, which were then arranged into groups of features with similar profiles using the Markov clustering algorithm (MCL) with the default inflation setting (2.2) for optimal clustering. Gene enrichment analysis was performed on lists of features chosen based on differential gene expression patterns using default settings of the FuncAssociate 2.0 web application [60]. Gene enrichment was calculated according to the significance (p< 0.05) of the association between the list of features and the GO attributes represented on the microarray. Relative expression ratios from RT-qPCR experiments were tested for normality and equal variance and log transformed to allow assumptions to be satisfied before being subjected to one way ANOVA using Minitab 16.1 software (Minitab Inc., UK). The significance level was set at p<0.05 in all tests.

## Competing interests

The authors declare that they have no competing interests.

## Authors’ contributions

SNC contributed to experimental design, performed the microarray design, experiments and data analysis and contributed to writing the manuscript with AS and JEB. JEB supervised and advised on microarray design and data analysis, contributed to planning and supervision of the study and assisted in writing of the manuscript. JBT contributed to experimental design and supervised microarray hybridisations and data extraction. JHI participated in performing the microarray hybridisations and advised on RT-qPCR validation of the experiments. MB contributed to the bioinformatic analysis of microarray experiments. STGB advised on experimental design and bioinformatic methods for gene expression data analysis. PJS contributed to planning and supervision of the study. AJN supervised subtracted library preparation and sequencing. KG contributed to experimental design and supervised the sequencing of subtracted libraries at the GenePool Genomics Facility. AS planned and supervised the study, supported the microarray data analysis and contributed to the preparation of the manuscript. All authors read and approved the final manuscript.

## Supplementary Material

Additional file 1 Enrichment of GO classes in the list of features showing differential expression between salmon louse strains. Click here for file

Additional file 2 **Genes identified from the features grouped in network cluster 1.** Changes in expression of genes in cluster 1 (Figure 5) observed following exposure of two salmon louse strains to EMB, expressed relative to gene expression in the matching solvent (SOL) control. Annotated genes (35%) are arranged by category of biological function. Features with identical annotation were removed prior to categorising biological function. Fold changes in expression ≥ 1.3 are underlined. Click here for file

Additional file 3 **Genes identified from the features grouped in network cluster 2.** Changes in expression of genes in cluster 2 (Figure 5) observed following exposure of two salmon louse strains to EMB, expressed relative to gene expression in the matching solvent (SOL) control. For further details, please see legend of additional file 2. Click here for file

Additional file 4 Enrichment of GO classes among features of network cluster 1. Click here for file

Additional file 5 Primers used for RT-qPCR analysis of relative gene expression between salmon louse strains (experiments 1 and 2). Click here for file

## References

[B1] ffrench-Constant RH Daborn PJ Le Goff G The genetics and genomics of insecticide resistance Trends Genet 2004 20 163 170 10.1016/j.tig.2004.01.003 15036810

[B2] Wolstenholme AJ Fairweather I Prichard R von Samson-Himmelstjerna G Sangster NC Drug resistance in veterinary helminths Trends Parasitol 2004 20 469 476 10.1016/j.pt.2004.07.010 15363440

[B3] Kabata Z Parasitic copepoda of British fishes 1979 London: British Museum (Natural History) for the Ray Society

[B4] Costello MJ The global economic cost of sea lice to the salmonid farming industry J Fish Dis 2009 32 115 118 10.1111/j.1365-2761.2008.01011.x 19245636

[B5] Denholm I Devine GJ Horsberg TE Sevatdal S Fallang A Nolan DV Powell R Analysis and management of resistance to chemotherapeutants in salmon lice, *Lepeophtheirus salmonis* (Copepoda: Caligidae) Pest Manag Sci 2002 58 528 536 10.1002/ps.482 12138619

[B6] Jones MW Sommerville C Wootten R Reduced sensitivity of the salmon louse, *Lepeophtheirus salmonis*, to the organophosphate dichlorvos J Fish Dis 1992 15 197 202 10.1111/j.1365-2761.1992.tb00654.x

[B7] Sevatdal S Horsberg TE Determination of reduced sensitivity in sea lice (*Lepeophtheirus salmonis* Kroyer) against the pyrethroid deltamethrin using bioassays and probit modelling Aquaculture 2003 218 21 31 10.1016/S0044-8486(02)00339-3

[B8] Treasurer JW Wadsworth S Grant A Resistance of sea lice, *Lepeophtheirus salmonis* (Kroyer), to hydrogen peroxide on farmed Atlantic salmon, Salmo salar L Aquacult Res 2000 31 855 860 10.1046/j.1365-2109.2000.00517.x

[B9] Heumann J Carmichael S Bron JE Tildesley A Sturm A Molecular cloning and characterisation of a novel P-glycoprotein in the salmon louse *Lepeophtheirus salmonis* Comparative Biochemistry and Physiology - C Toxicology and Pharmacology 2012 155 198 205 10.1016/j.cbpc.2011.08.004 21867772

[B10] Igboeli OO Fast MD Heumann J Burka JF Role of P-glycoprotein in emamectin benzoate (SLICE®) resistance in sea lice, *Lepeophtheirus salmonis* Aquaculture 2012 344–349 40 47

[B11] Stone J Sutherland IH Sommerville CS Richards RH Varma KJ The efficacy of emamectin benzoate as an oral treatment of sea lice, *Lepeophtheirus salmonis* (Kroyer), infestations in Atlantic salmon, *Salmo salar L* J Fish Dis 1999 22 261 270

[B12] Geary TG Ivermectin 20 years on: maturation of a wonder drug Trends Parasitol 2005 21 530 532 10.1016/j.pt.2005.08.014 16126457

[B13] McCavera S Walsh TK Wolstenholme AJ Nematode ligand-gated chloride channels: an appraisal of their involvement in macrocyclic lactone resistance and prospects for developing molecular markers Parasitology 2007 134 1111 1121 10.1017/S0031182007000042 17608971

[B14] Beech RN Skuce P Bartley DJ Martin RJ Prichard RK Gilleard JS Anthelmintic resistance: markers for resistance, or susceptibility? Parasitology 2011 138 160 174 10.1017/S0031182010001198 20825689 PMC3064440

[B15] Njue AI Prichard RK Genetic variability of glutamate-gated chloride channel genes in ivermectin-susceptible and -resistant strains of *Cooperia oncophora* Parasitology 2004 129 741 751 10.1017/S0031182004006183 15648697

[B16] McCavera S Rogers AT Yates DM Woods DJ Wolstenholme AJ An ivermectin-sensitive glutamate-gated chloride channel from the parasitic nematode *Haemonchus contortus* Mol Pharmacol 2009 75 1347 1355 10.1124/mol.108.053363 19336526 PMC2684884

[B17] Ardelli BF Prichard RK Identification of variant ABC-transporter genes among onchocerca volvulus collected from ivermectin-treated and untreated patients in Ghana, West Africa Ann Trop Med Parasitol 2004 98 371 384 10.1179/000349804225003415 15228718

[B18] James CE Davey MW Increased expression of ABC transport proteins is associated with ivermectin resistance in the model nematode *Caenorhabditis elegans* Int J Parasitol 2009 39 213 220 10.1016/j.ijpara.2008.06.009 18708066

[B19] Chen X Yuan L Du Y Zhang Y Wang J Cross-resistance and biochemical mechanisms of abamectin resistance in the western flower thrips, *Frankliniella occidentalis* Pesticide Biochemistry and Physiology 2011 101 34 38 10.1016/j.pestbp.2011.07.001

[B20] Tribble ND Burka JF Kibenge FSB Evidence for changes in the transcription levels of two putative P-glycoprotein genes in sea lice (*Lepeophtheirus salmonis*) in response to emamectin benzoate exposure Mol Biochem Parasitol 2007 153 59 65 10.1016/j.molbiopara.2007.02.002 17350696

[B21] Tribble ND Burka JF Kibenge FSB Identification of the genes encoding for putative gamma aminobutyric acid (GABA) and glutamate-gated chloride channel (GluCl) alpha receptor subunits in sea lice (*Lepeophtheirus salmonis*) J Vet Pharmacol Ther 2007 30 163 167 10.1111/j.1365-2885.2007.00823.x 17348903

[B22] Theocharidis A van Dongen S Enright AJ Freeman TC Network visualization and analysis of gene expression data using BioLayout express (3D) Nat Protoc 2009 4 1535 1550 10.1038/nprot.2009.177 19798086

[B23] Dent JA Smith MM Vassilatis DK Avery L The genetics of ivermectin resistance in *Caenorhabditis elegans* Proc Natl Acad Sci USA 2000 97 2674 2679 10.1073/pnas.97.6.2674 10716995 PMC15988

[B24] Kane NS Hirschberg B Qian S Hunt D Thomas B Brochu R Ludmerer SW Zheng YC Smith M Arena JP Cohen CJ Schmatz D Warmke J Cully DF Drug-resistant *Drosophil*a indicate glutamate-gated chloride channels are targets for the antiparasitics nodulisporic acid and ivermectin Proc Natl Acad Sci USA 2000 97 13949 13954 10.1073/pnas.240464697 11095718 PMC17681

[B25] Bloomquist JR Chloride channels as tools for developing selective insecticides Arch Insect Biochem Physiol 2003 54 145 156 10.1002/arch.10112 14635176

[B26] Feng XP Hayashi J Beech RN Prichard RK Study of the nematode putative GABA type-A receptor subunits: evidence for modulation by ivermectin J Neurochem 2002 83 870 878 10.1046/j.1471-4159.2002.01199.x 12421359

[B27] Krause RM Buisson B Bertrand S Corringer PJ Galzi JL Changeux JP Bertrand D Ivermectin: A positive allosteric effector of the α7 neuronal nicotinic acetylcholine receptor Mol Pharmacol 1998 53 283 294 9463487 10.1124/mol.53.2.283

[B28] Vogel C Marcotte EM Insights into the regulation of protein abundance from proteomic and transcriptomic analyses Nat Rev Genet 2012 13 227 232 22411467 10.1038/nrg3185 PMC3654667

[B29] Cully DF Vassilatis DK Liu KK Paress PS VanderPloeg LHT Schaeffer JM Arena JP Cloning of an avermectin sensitive glutamate-gated chloride channel from *Caenorhabditis elegans* Nature 1994 371 707 711 10.1038/371707a0 7935817

[B30] Cully DF Paress PS Liu KK Schaeffer JM Arena JP Identification of a drosophila melanogaster glutamate-gated chloride channel sensitive to the antiparasitic agent avermectin J Biol Chem 1996 271 20187 20191 10.1074/jbc.271.33.20187 8702744

[B31] Shan Q Haddrill JL Lynch JW Ivermectin, an unconventional agonist of the glycine receptor chloride channel J Biol Chem 2001 276 12556 12564 10.1074/jbc.M011264200 11278873

[B32] Silberberg SD Li M Swartz KJ Ivermectin interaction with transmembrane helices reveals widespread rearrangements during opening of P2X receptor channels Neuron 2007 54 263 274 10.1016/j.neuron.2007.03.020 17442247

[B33] Yusein S Velikova N Kupenova P Hardie R Wolstenholme A Semenov E Altered ivermectin pharmacology and defective visual system in Drosophila mutants for histamine receptor HCLB Invert Neurosci 2008 8 211 222 10.1007/s10158-008-0078-2 18839229

[B34] Rao VTS Siddiqui SZ Prichard RK Forrester SG A dopamine-gated ion channel (HcGGR3*) from *Haemonchus contortus* is expressed in the cervical papillae and is associated with macrocyclic lactone resistance Mol Biochem Parasitol 2009 166 54 61 10.1016/j.molbiopara.2009.02.011 19428673

[B35] Burridge L Weis JS Cabello F Pizarro J Bostick K Chemical use in salmon aquaculture: A review of current practices and possible environmental effects Aquaculture 2010 306 7 23 10.1016/j.aquaculture.2010.05.020

[B36] Labbé P Berticat C Berthomieu A Unal S Bernard C Weill M Lenormand T Forty years of erratic insecticide resistance evolution in the mosquito *Culex pipiens* PLoS Genet 2007 3 e205 10.1371/journal.pgen.0030205 18020711 PMC2077897

[B37] Nebert DW Gonzalez FJ P450 genes: structure, evolution, and regulation Annu Rev Biochem 1987 56 945 993 10.1146/annurev.bi.56.070187.004501 3304150

[B38] Bernhardt R Cytochrome P450: Structure, function, and generation of reactive oxygen species Rev Physiol Biochem Pharmacol 1995 127 137 221 8533008 10.1007/BFb0048267

[B39] Heckel DG Insecticide resistance after silent spring Science 2012 337 1612 1614 10.1126/science.1226994 23019637

[B40] Nelson DR Metazoan cytochrome P450 evolution Comp Biochem Physiol C Pharmacol Toxicol Endocrinol 1998 121 15 22 10.1016/S0742-8413(98)10027-0 9972448

[B41] Helvig C Koener JF Unnithan GC Feyereisen R CYP15A1, the cytochrome P450 that catalyzes epoxidation of methyl farnesoate to juvenile hormone III in cockroach *corpora allata* Proc Natl Acad Sci USA 2004 101 4024 4029 10.1073/pnas.0306980101 15024118 PMC384689

[B42] Guittard E Blais C Maria A Parvy JP Pasricha S Lumb C Lafont R Daborn PJ Dauphin-Villemant C CYP18A1, a key enzyme of Drosophila steroid hormone inactivation, is essential for metamorphosis Dev Biol 2011 349 35 45 10.1016/j.ydbio.2010.09.023 20932968

[B43] Baldwin WS Marko PB Nelson DR The cytochrome P450 (CYP) gene superfamily in *Daphnia pulex* BMC Genomics 2009 10 169 10.1186/1471-2164-10-169 19383150 PMC2678163

[B44] Dam E Rewitz KF Styrishave B Andersen O Cytochrome P450 expression is moult stage specific and regulated by ecdysteroids and xenobiotics in the crab *Carcinus maenas* Biochem Biophys Res Commun 2008 377 1135 1140 10.1016/j.bbrc.2008.10.125 18983823

[B45] Jones PM O'Mara ML George AM ABC transporters: a riddle wrapped in a mystery inside an enigma Trends Biochem Sci 2009 34 520 531 10.1016/j.tibs.2009.06.004 19748784

[B46] Leslie EM Deeley RG Cole SPC Multidrug resistance proteins: role of P-glycoprotein, MRP1, MRP2, and BCRP (ABCG2) in tissue defense Toxicol Appl Pharmacol 2005 204 216 237 10.1016/j.taap.2004.10.012 15845415

[B47] Bain LJ LeBlanc GA Interaction of structurally diverse pesticides with the human MDR1 gene product P-glycoprotein Toxicol Appl Pharmacol 1996 141 288 298 8917702 10.1006/taap.1996.0286

[B48] Schinkel AH Smit JJM Van Tellingen O Beijnen JH Wagenaar E Van Deemter L Mol CAAM Van Der Valk MA Robanus-Maandag EC Te Riele HPJ Berns AJM Borst P Disruption of the mouse mdr1a P-glycoprotein gene leads to a deficiency in the blood–brain barrier and to increased sensitivity to drugs Cell 1994 77 491 502 10.1016/0092-8674(94)90212-7 7910522

[B49] Lanning CL Ayad HM Abou-Donia MB P-glycoprotein involvement in cuticular penetration of [14C]thiodicarb in resistant tobacco budworms Toxicol Lett 1996 85 127 133 10.1016/0378-4274(96)03654-5 8644124

[B50] Xu M Molento M Blackhall W Ribeiro P Beech R Prichard R Ivermectin resistance in nematodes may be caused by alteration of P-glycoprotein homolog Mol Biochem Parasitol 1998 91 327 335 10.1016/S0166-6851(97)00215-6 9566525

[B51] Ardelli BF Guerriero SB Prichard RK Ivermectin imposes selection pressure on P-glycoprotein from *Onchocerca volvulus*: Linkage disequilibrium and genotype diversity Parasitology 2006 132 375 386 16280093 10.1017/S0031182005008991

[B52] Sevatdal S Copley L Wallace C Jackson D Horsberg TE Monitoring of the sensitivity of sea lice (*Lepeophtheirus salmonis*) to pyrethroids in Norway, Ireland and Scotland using bioassays and probit modelling Aquaculture 2005 244 19 27 10.1016/j.aquaculture.2004.11.009

[B53] Helgesen KO Horsberg TE Influence of different materials on the concentration of delousing agents in sea water during bioassays J Fish Dis 2013 36 529 532 10.1111/jfd.12046 23163800

[B54] Chomczynski P Mackey K Modification of the TRI-reagent procedure for isolation of RNA from polysaccharide-rich and proteoglycan-rich sources Biotechniques 1995 19 942 945 8747660

[B55] Yasuike M Leong J Jantzen SG von Schalburg KR Nilsen F Jones SRM Koop BF Genomic resources for sea lice: analysis of ESTs and mitochondrial genomes Marine Biotechnol 2012 14 155 166 10.1007/s10126-011-9398-z PMC3280385 21748342

[B56] Morais S Pratoomyot J Taggart JB Bron JE Guy DR Bell JG Tocher DR Genotype-specific responses in Atlantic salmon (*Salmo salar*) subject to dietary fish oil replacement by vegetable oil: a liver transcriptomic analysis BMC Genomics 2011 12 255 10.1186/1471-2164-12-255 21599965 PMC3113789

[B57] Pfaffl MW Tichopad A Prgomet C Neuvians TP Determination of stable housekeeping genes, differentially regulated target genes and sample integrity: BestKeeper - excel-based tool using pair-wise correlations Biotechnol Lett 2004 26 509 515 15127793 10.1023/b:bile.0000019559.84305.47

[B58] Pfaffl MW A new mathematical model for relative quantification in real-time RT-PCR Nucleic Acids Res 2001 29 e45 10.1093/nar/29.9.e45 11328886 PMC55695

[B59] Leaver MJ Villeneuve LAN Obach A Jensen L Bron JE Tocher DR Taggart JB Functional genomics reveals increases in cholesterol biosynthetic genes and highly unsaturated fatty acid biosynthesis after dietary substitution of fish oil with vegetable oils in Atlantic salmon (*Salmo salar*) BMC Genomics 2008 9 299 10.1186/1471-2164-9-299 18577222 PMC2459193

[B60] Berriz GF Beaver JE Cenik C Tasan M Roth FP Next generation software for functional trend analysis Bioinformatics 2009 25 3043 3044 10.1093/bioinformatics/btp498 19717575 PMC2800365

